# Early detection of COPD patients in GOLD 0 population: an observational non-interventional cohort study - MARKO study

**DOI:** 10.1186/s12890-017-0378-6

**Published:** 2017-02-10

**Authors:** Žarko Vrbica, Marina Labor, Ivan Gudelj, Slavica Labor, Iva Jurić, Davor Plavec, for the MARKO study group

**Affiliations:** 1 Department of Pulmonology and Immunology, General Hospital Dubrovnik, Dr. Roka Mišetića 2, Dubrovnik, Croatia; 2 University of Dubrovnik, Branitelja Dubrovnika 29, Dubrovnik, Croatia; 3 Department of Pulmonology, University Hospital Center Osijek, Josipa Huttlera 4, Osijek, Croatia; 4 Faculty of Medicine, J.J. Strossmayer University of Osijek, Ulica cara Hadrijana 10E, Osijek, Croatia; 5 Department of Pulmonology, University Hospital Center Split, Spinčićeva 1, Split, Croatia; 6 Department of Internal Medicine, University Hospital Center Osijek, Josipa Huttlera 4, Osijek, Croatia; 7 Research Department, Children’s Hospital Srebrnjak, Srebrnjak 100, Zagreb, Croatia

**Keywords:** Biological markers, Chronic obstructive pulmonary disease (COPD), Cigarette smoking, Disease susceptibility, Early diagnosis

## Abstract

**Background:**

Main risk factor for the development of chronic obstructive pulmonary disease (COPD) is smoking, although only less than 1/3 of smokers develop clinically manifest COPD. COPD’s progressive nature with high disability and mortality makes it plausible to detect it as early as possible thus allowing for an early intervention. The only tool for an early diagnosis that could be used on the global scale is spirometry, even though symptoms and deprivation of health related quality of life (HRQoL) precede relevant spirometric changes. Existing HRQoL questionnaires are too complicated or not developed for an early detection of COPD. The aim of our study was to develop a new simple HRQoL tool that will allow (alone or in combination with other markers) early detection of patients with COPD.

**Methods:**

A multicenter prospective cohort study recruiting 500 subjects at risk for COPD (smokers/ex-smokers ≥20 pack-years, 40–65 years, both sexes, with no prior diagnosis of COPD) will be carried out in two phases: (1) cross-sectional - development and validation of a new questionnaire; and (2) prospective - follow-up of a cohort of patients at risk for COPD. Subjects were recruited by 25 GPs and assessed for COPD by dedicated pulmonologists in 7 hospital centers using a predefined protocol: HRQoL, history, physical, blood sampling, exhaled breath temperature (EBT), lung function, 6-min walk test (6MWT). Patients without COPD and those in GOLD stage 1 at initial assessment will be reassessed for disease progression by the same pulmonologist after 2 and 5 years.

**Discussion:**

This is one of the first cohort studies attempting to establish the incidence of COPD in the pre-symptomatic stage before significant end organ damage. We intend to assess the validity, predictability and discriminative power (‘healthy’ smokers vs. pre-symptomatic phase in newly developed COPD) of newly developed HRQoL tool alone or in combination with other markers; EBT, lung function, 6MWT, genomics, transcriptomics, proteomics). We expect that the results of this study can improve our understanding of the development of COPD, identify some new underlying pathophysiological pathways, and offer to sensitive smokers/ex-smokers new preventive and early intervention measures thus improving the management of COPD.

**Trial registration:**

Clinicaltrial.gov NCT01550679 retrospectively registered February 28, 2012.

## Background

Chronic obstructive pulmonary disease (COPD) is a major cause of morbidity and mortality globally, responsible for approximately 5 million deaths every year, with expected significant rise in mortality untill the year 2020. COPD mortality is low at age 45, but significantly rises after the age of 65 with significant comorbidity already present. COPD has a major economical and public health impact especially with regard to severe disease and exacerbations. Major risk factor for COPD development is tobacco smoke, although only less than 1/3 of smokers develop COPD during their lifetime. Because of its progressive nature COPD ends with early disability and mortality. Stopping or slowing down the progression of the disease is still an unmet need, although therapeutic interventions in COPD patients have shown to have significantly larger impact if they were started earlier in the course of the disease [1–4]. An early diagnosis should allow for an early intervention, thus possibly preventing the progression of COPD, alleviating symptoms and improving general wellbeing, preventing complications and comorbidities and early mortality [5]. The only available method for an early diagnosis that is suitable for screening purposes is spirometry (also used as the ‘gold standard’ for COPD diagnosis). Although screening on a global scale is not recommended by COPD guidelines, recommendations for early diagnosis (case finding) have been heavily advocated [6, 7]. New insights of the genetic background of COPD development have been revealed from cross-sectional large scale genetic studies but these data still need confirmation on a global scale using prospective data [8]. On the other hand, symptoms and loss in health related quality of life (HRQoL) often precede diagnostically relevant loss of lung function (FEV_1_/FVC <0.7 or < lower limit of normal [LLN]) [6]. Consequently we need new simple tools to detect patients in an early (pre)symptomatic stage of COPD before significant end organ damage. Based on these assumptions; the possibility of identifying the population at risk and the possibility of producing significant benefit for the patient if COPD is identified early, we decided to develop and test a new tool (to be used alone and in combination with other markers) in a prospective cohort study in a population at risk (smokers/ex-smokers with significant cumulative exposure to tobacco smoke, with no prior diagnosis of COPD). This new tool should be based on a prerequisite for a screening tool; usable on a global level, cheap, self-applicable, moderate to high sensitivity and high specificity (no false positives) for COPD. As existing HRQoL questionnaires are too complicated (Chronic Respiratory Questionnaire - CRQ, St. George Respiratory Questionnaire - SGRQ), and/or not developed for the purpose of early detection of COPD (Clinical COPD Questionnaire - CCQ, COPD Assessment Test - CAT), we have constructed, developed, and intend to validate a simple self-applicable questionnaire to detect early changes in HRQoL related to future COPD development [9–13].

## Methods

### The aim, design and setting of the study

The aim of this two phase prospective observational cohort study in subjects at risk for COPD was to construct, develop and validate a new tool (newly constructed self-applicable HRQoL questionnaire – MARKO questionnaire) to be used alone or in combination with other markers (exhaled breath temperature [EBT], lung function, inflammatory markers, and omics) in order to identify subjects who will develop COPD, even before significant end organ damage that can be identified using spirometry.

The MARKO study is a prospective, observational, non-interventional cohort study of subjects (both sexes) at risk for the development of COPD (smokers/ex-smokers with a smoking history of ≥20 pack-years), without a previous diagnosis of COPD. The study for all investigational sites was approved by the Children’s Hospital Srebrnjak Ethics Committee and performed in accordance with the Declaration of Helsinki, good clinical practice, and all relevant international and national legislations. According to a national legislation regarding non-interventional studies an approval from a single local ethics committee meets the ethical requirements for such a study. The MARKO study has been carried out with the cooperation of 25 general practitioners (GPs) and 7 tertiary hospital research centers (within Departments of Pulmonology) since 2010 (Table 1).

**Table 1:** The list of study sites and researchers involved in MARKO study (MARKO study group)

Study sites	Contact person/researcher	Role
Children’s Hospital Srebrnjak, Zagreb, Croatia	Assoc.Prof. Davor Plavec, MD, MSc, PhD	Principal Investigator
General Hospital Dubrovnik, Dubrovnik, Croatia	Žarko Vrbica, MD, MSc	Principal Investigator
University of Liverpool, Liverpool, UK	Prof. Peter MA Calverley, MD, PhD	Study consultant
University of Zagreb Centre for Croatian Studies, Zagreb, Croatia	Assist.Prof. Adrijana Košćec Đuknić, PhD	Psychologist
Institute for Medical Research and Occupational Health, Zagreb, Croatia	Assist.Prof. Biserka Radošević-Vidaček, BA, MA, PhD	Psychologist
University Hospital Center Osijek, Osijek, Croatia	Marina Labor, MD, PhD	Co-Investigator
	Slavica Labor, MD, PhD	Subinvestigator
	Iva Jurić, MD	Subinvestigator
University Hospital Center Split, Split, Croatia	Assist.Prof. Ivan Gudelj, MD, PhD	Co-Investigator
Univerity Hospital Center Rijeka, Rijeka, Croatia	Assist.Prof. Ljiljana Bulat Kardum, MD, PhD	Co-Investigator
University Hospital Dubrava, Zagreb, Croatia	Assoc.Prof. Neven Tudorić, MD, PhD	Co-Investigator
	Đivo Ljubičić, MD	Subinvestigator
University Hospital Center Zagreb, Zagreb, Croatia	Assoc.Prof. Sanja Popović Grle, MD, PhD	Subinvestigator
Special Hospital for Lung Diseases, Zagreb, Croatia	Tajana Jalušić Glunčić, MD	Subinvestigator
	Tina Lukić, MD	Subinvestigator
General practice offices, Osijek, Croatia	Albina Dumić, MD, PhD	
	Renata Grgurić, MD	
	Ljiljana Ismić, MD	
	Monika Jeđud, MD	
	Jasna Nagyszombaty-Šarić, MD	
	Darja Nelken-Bestvina, MD	
	Sanda Pribić, MD	
	Alen Stojanović, MD	
	Bojana Vakanjac, MD	
General Practice offices, Split, Croatia	Merim Bezdrov, MD	
	Mirjana Bezdrov, MD	
	Ivana Boban, MD	
	Jadranka Radman, MD	
	Rosanda Rosandić Piasevoli, MD	
	Vanja Viali, MD	
General Practice offices, Zagreb, Croatia	Vjekoslava Amerl-Šakić, MD	
	Ljiljana Lulić-Karapetrić, MD	
	Suzana Maltar-Delija, MD	
	Dubravka Margaretić, MD	
	Davorka Martinković, MD	
	Zdenka Meštrović, MD	
General Practice office, Donji Muć, Croatia	Karla Tudja, MD	
General Practice office, Postira, Croatia	Nataša Mrduljaš-Đuić, MD	
General practice office, Rijeka, Croatia	Assoc.Prof. Ines Diminić Lisica, MD, PhD	
General Practice office, Strmec Samoborski, Croatia	Ines Balint, MD	

*Primary endpoint:* to develop and validate the MARKO questionnaire to be used alone or together with other markers to identify subjects that will develop COPD.

*Secondary endpoints:* (a) to evaluate psychometric characteristics of the MARKO questionnaire; (b) to evaluate if the developed screening questionnaire together with the COPD-6^TM^ measurement discriminates COPD patients with GOLD stage 0 (symptomatic smokers) and GOLD 1 from ‘healthy’ smokers and patients with COPD GOLD 2 or higher based on the evaluation made by the pulmonologist; (c) to determine the rate of progression of COPD in patients with GOLD 0 during the follow-up; (d) to determine the prevalence of concomitant disorders in this population; (e) to identify diagnostic parameters that are most sensitive for early impairment in COPD (best discriminate between COPD GOLD 0 and GOLD 1); (f) to compare the MARKO questionnaire with diagnostic tools used for evaluation of patients (SGRQ, CAT, history, physical, lung function, 6MWT); (g) to assess the prevalence of different stages of COPD (specifically GOLD 0 and GOLD 1) in the population at risk for COPD and in the general population.

**Fig. 1:**
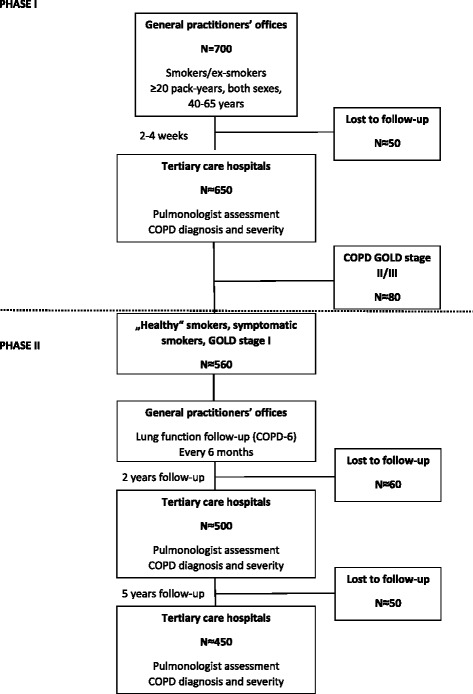
Flow chart of subjects through the MARKO study

MARKO is a two phase study: Phase I - a cross-sectional; Phase II - prospective, observational, non-interventional cohort study. Flow diagram of subjects through the MARKO study is presented in Fig. 1 and all the assessments during the study are summarized in Table 2.

**Table 2:** List of subjects’ assessment through different stages of MARKO study

Assessment	Time/place						
	0	2-4	6	12	18	24	60
		w	m	m	m	m	m
	GP	P	GP	GP	GP	P	P
Prescreening	X						
Informed consent	X						
Inclusion/exclusion criteria	X						
MARKO questionnaire	X	X				X	X
CAT		X				X	X
SGRQ		X				X	X
History		X				X	X
Physical		X				X	X
EBT before and after cigarette		X					
Lung function							
COPD-6™	X	X	X	X	X		
Spirometry with bronchodilator test		X				X	X
Impulse oscilometry^a^		X					
Lung diffusion capacity		X					
Body plethysmography^a^		X					
Blood sampling (hematology; highly sensitive C-reactive protein; blood							
stored for DNA, RNA, plasma and serum)^b^		X					
Functional exercise capacity (6-minute walk test)		X					
COPD diagnosis		X				X	X

Legend: w - week, m - month, GP - general practitioner’s office, P - pulmonologist at tertiary care hospital, CAT - COPD Assessment Test, SGRQ - St. George Respiratory Questionnaire, EBT - exhaled breath temperature

^a^Impulse oscilometry and body plethysmography was done only in one center

^b^Blood sampling was done only in subjects that signed additional Informed consent

In Phase I up to 700 subjects will be recruited with the risk of COPD by 25 GP’s offices (representing 25 GPs) in and around 4 major cities. Patients were approached by their GP during any (unrelated to respiratory problems) visit to the GP’s office if they were smokers or ex-smokers of the predefined age group for the study and were prescreened for inclusion/exclusion criteria using a structured interview. Eligible patients were given the Informed consent document with enough time to read it and to discuss any relevant issues regarding the study before consenting. All participants signed written consent for the follow-up study, and separately for bio-banking of blood for omics (DNA, RNA, serum and plasma) before taking part in the study and before any procedure was done. Inclusion criteria for the study were defined as: written consent; smokers/ex-smokers; both sexes; aged 40–65 years at inclusion; smoking history of at least 20 pack-years (calculated as a number of cigarettes smoked per day multiplied by the number of years of smoking divided by 20); and no previous diagnosis of COPD. Exclusion criteria were defined as: any clinically relevant chronic disease (cardiovascular, cerebrovascular, diabetes, hepatitis, nephropathy, chronic dialysis, systemic disorder, cancer) significantly affecting HRQoL; ongoing immunosuppressive therapy; preceding acute respiratory disease 4 weeks before inclusion; hospitalization for any reason during the past 3 months; myocardial infarction (MI), cerebrovascular infarction (CVI) or transient ischemic attack (TIA) during the past 6 months; diagnosis of asthma; and an inability to perform the diagnostic protocol. Exclusion criteria were introduced in order not to exclude patients with comorbidities common in COPD, but to exclude those that represent acute/subacute clinical states/disorders or states of recovery from major clinical disorders representing an absolute or relative contraindication for spirometry or significantly influencing the diagnostic process or an already present diagnosis of respiratory disorder (e.g., asthma). After inclusion patients fill out the self-applicable MARKO questionnaire in the GP’s office and measurement of lung function is done using COPD-6™ (4000 COPD-6™ Respiratory Monitor, Vitalograph Ltd., Buckingham, UK) recording the values of forced expiratory volume in 1 s (FEV_1_), forced expiratory volume in 6 s (FEV_6_ - as a surrogate marker of forced vital capacity [FVC]), FEV_1_/FEV_6_ (as a surrogate marker of FEV_1_/FVC), and lung age. Two to 4 weeks after inclusion eligible patients are referred to one of the 7 tertiary hospital research centers, to a designated team consisting of a pulmonologist, research nurse and lung function laboratory technician where a structured diagnostic workup consisting of the MARKO questionnaire, HRQoL questionnaires (SGRQ and CAT), structured history and physical, exhaled breath temperature (EBT) before (EBTb) and after a smoked cigarette (EBTc), lung function testing with bronchodilator (salbutamol), impulse oscilometry (only in one center), lung diffusion capacity (DL_CO_), body plethysmography (only in one center), blood sampling (hematology; highly sensitive C-reactive protein [hs-CRP]; blood for DNA, RNA, plasma and serum), functional exercise capacity using 6-min walk test (6MWT), ending with the assessment for diagnosis and severity of COPD according to the Global Initiative for Chronic Obstructive Lung Disease (GOLD) and according to the American Thoracic Society/European Respiratory Society (ATS/ERS) recommendation for airway limitation using lower level of normal (LLN) [6, 14].

In Phase II up to 500 subjects will be recruited from Phase I, assessed by designated pulmonologists as ‘healthy’ smokers, symptomatic smokers (GOLD 0) and as COPD GOLD 1. Selected subjects will be followed for at least 5 years; first assessment after 2 years (±2 months) and the second 5 years (±2 months) after the baseline. During the first 2 years of follow-up all subjects will have regular lung function measurement using COPD-6^TM^ at their GP’s office in 6 month intervals. All subjects will be assessed by the same pulmonologist for diagnosis and severity of COPD according to GOLD and ATS/ERS after 2 and 5 years for the progression of disease defined by three outcome measures: (1) newly diagnosed COPD (ND COPD); (2) disease progression (DP; newly diagnosed COPD + progression to higher severity stage); and (3) high rate of loss of lung function (LoLF; >70 mL/year for post bronchodilator FEV_1_). Incidence of newly diagnosed COPD after 2- and 5-years of follow up will be used to identify diagnostic parameters that are most sensitive for early impairment in COPD, to determine the predictability of developed screening MARKO questionnaire alone or with other markers of early impairment in COPD. Blood samples from a subsample of patients who give the consent for omics research will be used to develop omics markers predictive for the future development of COPD and disease progression.

This research will attempt to answer to following questions: (1) can we identify cheap and simple tools that will allow a precise and accurate identification of subjects from a population at risk of developing COPD in the future; (2) is there a combination (pattern) of tools, functional parameters, genetic and biochemical markers that can reliably predict the development of COPD in a population at risk, thus allowing early intervention.

### Methods

#### Screening questionnaire (MARKO questionnaire)

The MARKO questionnaire is a newly constructed HRQoL questionnaire developed by a group of experts; three experienced pulmonologists (ZV, DP, PMAC) and two psychologists (BRV and AKD). The questionnaire comprises 18 questions covering the manifestation and frequency of the symptoms already present at early stages of COPD that could impact the HRQoL in patients. The participants were asked to rate the frequency of their symptoms over a designated period of time (e.g., over the past three months for coughing, shortness of breath, expectoration, and over the past 12 months for pulmonary infections). They also rated their breathing quality and general health status. Furthermore, they reported on shortness of breath during daily life activities requiring different physical strain, and compared their physical abilities and fatigue with respect to their referential age group. The total scores ranged from 0 to 57 points, where the higher scores indicated poorer HRQoL.

### Other HRQoL questionnaires

HRQoL will be additionally assessed using 2 standard questionnaires; St. George Respiratory Questionnaire (SGRQ) and COPD Assessment Test (CAT). The SGRQ is a standardized self-administered airways disease-specific questionnaire divided into three subscales: symptoms (8 items), activity (16 items), and impacts (26 items). SGRQ scores were calculated using score calculation algorithms and missing data imputation (if the total number of missing items was ≤10) using the Excel® SGRQ calculator. For each subscale and for the overall questionnaire, scores range from zero (no impairment) to 100 (maximum impairment) [10, 11]. The CAT is a validated, short (8-item) and simple patient completed questionnaire, with good discriminant properties, developed for use in routine clinical practice to measure the health status of patients with COPD. Every item has a scale of 0–5 so the scoring range is from zero (no impairment) to 40 (maximum impairment) [13]. Both HRQoL questionnaires were self-completed by patients before any other procedure was done and after the MARKO questionnaire.

### Lung function

Spirometry was performed using computerized pneumotachographs (Jaeger®, CareFusion, CA, USA) using the same procedure at all clinical sites (lung function labs at tertiary hospitals) in agreement with the ATS/ERS standardization [15]. The best of three technically satisfactory efforts was recorded. Bronchodilator test was done with repeated spirometry 20 min after inhalation of 400 mcg of salbutamol using the inhalation chamber. Spirometric parameters (FVC, FEV_1_, FEV_1_/FVC ratio, peak expiratory flow [PEF], forced expiratory flow between 25 and 75% FVC [FEF_25–75_]) were recorded as absolute values and as percentage of predicted according to Quanjer [16].

Impulse oscillometry (IOS) was done using Jaeger MasterScreen IOS (Viasys Healthcare, Inc., Yorba Linda, USA) for measurements of respiratory system impedance according to ATS/ERS recommendations and the following IOS data were collected: resistance of the respiratory system at 5 Hz (R_5_) and 20 Hz (R_20_) and reactance at 5 Hz (X_5_) [17]. Results were analyzed as absolute values and as percentages of predicted values (%) according to reference equations provided by the manufacturer [18].

Lung volume studies were carried out using a body-plethysmograph (Ganzhorn, Germany) according to ATS/ERS recommendations [19]. The following parameters were analyzed: airway resistance (RAW), expiratory airway resistance (RAWex), total lung capacity (TLC), residual volume (RV) and the RV/TLC ratio and expressed as percentages of predicted values according to ATS/ERS recommendations [19, 20].

Single-breath diffusing capacity of the lung for carbon monoxide (DL_CO_) was measured using a rapid carbon monoxide and helium analyzer (Ganzhorn, Germany), which was calibrated prior to each measurement. Values for DL_CO_ and DL_CO_ corrected for alveolar volume (V_A_) [DL_CO_/V_A_] were obtained and are reported as percent predicted values [21].

### Exhaled breath temperature

EBT was measured using X-Halo® device (Delmedica Investments, Singapore) according to previously validated method [22]. Patients were requested to inhale freely through the nose and to exhale through the mouth into the device at a rate and depth typical of their normal tidal breathing pattern. The maneuver was continued until the software of the instrument indicated that the measured value was stable, thus fulfilling the criteria of a previously described mathematical model. The tests were carried out at room temperature of 19–25 °C, and at relative humidity of 30–60% in the lung function lab where the atmosphere is controlled and measured. EBT was measured twice on the same occasion (during the initial visit); (1) baseline measurement before any other procedure (lung function, bronchodilator test, 6MWT) at least 1 h after the last smoked cigarette (EBTb) and (2) done only in active smokers 15 min after a smoked cigarette (EBTc) and recorded with precision of 1/100 of a °C. No other procedure apart from cigarette smoking was carried out between the two EBT measurements.

### Blood sampling and storage

Before initializing the sample collection process, it was important to ensure the correct order of blood collection. The PAXgene blood RNA tube had to be the last tube drawn in the study procedure process. Before sample collection, all tubes were labeled with patient identification number. Blood samples for serum were drawn at minimal volume of 3 ml in serum separation vacuum tubes (containing Z Serum Clot Activator gel) and sent to the laboratory. Samples were kept at room temperature for at least 30 min, but had to be processed within 2 h of blood collection. Upon centrifugation at 3000 rpm for 10 min, a minimum of 300 μl of serum was separated for further detection of hs-CRP level. The rest of the serum sample from every subject was transferred to a cryotube vial and stored at −20 °C.

Blood samples for plasma were drawn at minimal volume of 3 ml in K2EDTA vacuum blood collection tubes and were inverted several times to ensure proper mixing of additive with blood. Samples were sent to the laboratory where 200 μl of blood was separated for the purpose of complete blood cell hematological analysis. The remaining blood volume was kept at room temperature for at least 30 min, but had to be processed within 2 h of blood collection. Samples were then centrifuged at 3500 rpm for 10 min. The plasma sample from every subject was transferred to a cryotube vial and stored at −20 °C.

Blood samples for DNA extraction were drawn at minimal volume of 2 ml in K2EDTA vacuum blood collection tubes and were inverted several times to ensure proper mixing of the additive with blood. Samples were sent to the laboratory where they were kept at room temperature for 30 min, and inverted again before their transfer to a cryotube vial which was stored at −20 °C.

Blood samples for RNA extraction were drawn at volume of 2.5 ml in PAXgene blood RNA tubes, which were kept at room temperature prior to use. After blood collection, the PAXgene blood RNA tubes were immediately gently inverted 8–10 times to ensure proper mixing of additive with blood, after which they were stored at −20 °C.

Laboratory analyses were done in local laboratories and included complete blood count, white blood cells differential count, hematocrit, hemoglobin and hs-CRP.

Functional exercise capacity was assessed using 6MWT according to the ATS guidelines and expressed as walked distance in meters and as % of predicted according to Trooster et al. [23, 24].

### Data analyses

Data analyses are envisaged using STATISTICA version 12 (StatSoft, Inc., OK, USA), MedCalc Statistical Software version 16.8.4 (MedCalc Software bvba, Ostend, Belgium; https://www.medcalc.org; 2015) and RUMM2030 (RUMM Laboratory, Perth, Australia). Sample size calculation was done based on the following assumptions: we expected to find 25% patients in different stages of COPD according to the airflow limitation. We therefore expected that the sample would consist of 75% of ‘healthy’ and symptomatic smokers, 12.5% patients with COPD GOLD 1, 6.25% in GOLD 2 and 6.25% in GOLD 3 or 4 stages at initial visit with the expected difference in the MARKO questionnaire scores of 2 points and SD od 2.5 points between ‘healthy’ smokers vs. symptomatic smokers vs. COPD GOLD 1/2 having a statistical power of >80% with alpha = 0.05 for the sample size of at least 500 subjects. The expected yearly incidence rate for GOLD 1 and progression from GOLD 1 to GOLD 2 is planned at 10/per 100 patient-years.

Phase I data analysis will primarily focus on primary validation of the MARKO questionnaire (its psychometric characteristics), convergent and discriminant validity comparing data between different subgroups and with other HRQoL questionnaires (CAT and SGRQ). Categorical data will be compared between subgroups using chi-square test or Fisher exact test and continuous variables using Mann-Whitney *U*-test and Kruskal-Wallis ANOVA (non-normal distribution expected). Metric characteristics of the MARKO questionnaire will also be analyzed using Cronbach’s alpha, Lin’s concordance and Spearman’s correlation coefficients, analyzing inner consistency, test-retest reliability, and association with other measures of HRQoL and health status.

Phase II data analysis will focus on secondary validation of the MARKO questionnaire to determine the construct validity and predictability (alone or in combination with other markers) for future development and/or progression of COPD. Construct validity of the MARKO questionnaire will be assessed using factorial analysis to confirm the number of factors the questionnaire is measuring with calculations of inter- and intracorrelations between the factors and items. Primary objective will be to create a questionnaire with the smallest number of items with reliable predictive properties. Identifying items for potential deletion will be based on a hierarchical process: age and sex bias, floor and ceiling effects, item to total correlation and tests of redundancy (inter-item correlation). Rasch analysis will be used to identify items with the best fit to a predictive model. Utility of different markers for disease progression will be assessed using generalized linear/nonlinear regression models and expressed as odds ratio (OR) with 95% confidence intervals (CIs). Predictive power for the models will be presented using receiver operator curve (ROC) analysis with AUC (with 95% CIs) together with associated criterion, sensitivity, specificity and positive (PPV) and negative predictive (NPV) values. *P* < 0.05 will be used as statistically significant for all analyses with correction for multiple comparisons.

## Discussion

This is one of the first cohort studies attempting to establish the predictors and incidence of COPD in pre-symptomatic stage before clinical diagnosis and clinically documented end organ damage. Having in mind the progressive nature of COPD with high morbidity and early mortality, our aim is to try to develop and identify simple tools and markers or a pattern of the afore mentioned in order to allow diagnosis as early as possible, before a significant fall in lung function. The problem when using lung function for diagnosing COPD lies in the significant variability of what represents normal (reference) values and the need for a significant expertise in providing technically appropriate measurement, thus limiting its use for screening purposes or making an early diagnosis [13, 14, 25]. Numerous studies have been focused on testing simple screening tools (questionnaires and/or simplified flow measurement devices) for already developed COPD, in order to surpass the limitations of spirometry obtaining a specificity of up to 84.4%, with inadequate blinding between an index test and spirometry being a major source of bias in these studies [26]. We have managed to surpass this major bias using the subsample from Phase I of the MARKO study and showing that lung function testing with COPD-6™ can substitute spirometry testing in cases where it is not readily available to the patient/physician, but bearing in mind that the traditional cutoff value of <0.7 for FEV_1_/FEV_6_ ratio, cannot be the only criterion for COPD diagnosis and/or further referral [27]. Early diagnosis and early intervention (based on data from pathophysiological studies) is highly advocated and studies already starting chronic bronchodilator treatment in early stages of COPD (in GOLD stages 1 and 2) have been initiated [7, 28–30]. In order for it to be possible to intervene even earlier in the course of disease, with intervention being specific (not harmful), it is very important for method(s) to be developed to be highly specific in identifying subjects that will develop COPD. Genetic testing in studies like UK BiLEVE, using large population samples, showed that contrary to previous genetic studies significant genetic signals predictive for COPD irrespective of smoking can be picked up, identifying specific mechanisms underlying airflow limitation [8]. However these data should be further confirmed using other populations and follow-up.

Phase I of the MARKO study will be used to test the psychometric characteristics of the MARKO questionnaire; to evaluate discriminative power of the MARKO questionnaire together with COPD-6™ measurements between different developmental stages of COPD; to identify diagnostic parameters that are most sensitive for early impairment in COPD (discriminate between COPD GOLD 0 and 1); to compare the MARKO questionnaire with other measures used to evaluate patients (HRQoL, history, physical, lung function, functional capacity); and to assess the prevalence of different stages of COPD (specifically GOLD 0 and 1) in the population at risk for COPD. Preliminary data from Phase I of initial validation of the MARKO questionnaire showed the potential for the MARKO questionnaire to assess early health status changes in smokers at risk for chronic obstructive pulmonary disease [31]. Although exclusion criteria were introduced to exclude subjects with acute/subacute clinical disorders or states of recovery from major clinical disorders representing an absolute or relative contraindication for spirometry or significantly influencing the diagnostic process, the data analysis showed that more than half (56.3%) of the recruited subjects had one or more (up to 3) comorbidities (28.6% hypertension, 10% dyslipidemia, 5.2% diabetes and 4.9% peptic syndrome, etc.) [31].

Phase II of the MARKO study will be used to test the primary endpoint of the study: to validate the MARKO questionnaire as a tool to be used alone or combined with other markers in identifying subjects at risk (smokers/ex-smokers with relevant exposure to tobacco smoke) who will develop COPD during the follow-up. Based on previously published data that EBT could be a sensitive marker of airways inflammation [21] we introduced it as a possible predictive marker for future COPD. Recently published data from Carpagnano GE et al., and from our group (preliminary data from the MARKO study) showed the sensitivity of EBT to cigarette smoke and the potential to predict future development of COPD in current smokers [32, 33]. All other parameters were measured/gathered at the start of the study for a detailed phenotype/endotype of recruited subjects and as possible predictive markers for future COPD.

We anticipate that the MARKO study will give us answers regarding the predictability and discriminative power of a newly developed HRQoL tool (MARKO questionnaire) alone; or in combination with other markers, such as EBT (using new protocol of assessment) [33], lung function, 6MWT, genomics, transcriptomics, proteomics. We believe that the results of this study will improve our understanding of the development of COPD, identify some underlying pathophysiological pathways, and offer to sensitive smokers/ex-smokers the possibility of an earlier intervention, thus improving the management of COPD.

## Abbreviations

6MWT: 6-min walk test
ANOVA: Analysis of variance
ATS: American Thoracic Society
AUC: Area under the curve
CAT: COPD Assessment Test
CCQ: Clinical COPD Questionnaire
CI: Confidence interval
COPD: Chronic obstructive pulmonary disease
CRP: C reactive protein
CRQ: Chronic Respiratory Questionnaire
CVI: Cerebrovascular infarction
DL_CO_: Diffusing capacity of the lung for carbon monoxide
DP: Disease progression
EBT: Exhaled breath temperature
EBTb: Baseline EBT
EBTc: EBT after smoked cigarette
ERS: European Respiratory Society
FEF_25–75_: Forced expiratory flow between 25 and 75% FVC
FEV_1_: Forced expiratory volume in 1. second
FVC: Forced vital capacity
GCP: Good Clinical Practice
GOLD: Global initiative for chronic Obstructive Lung Disease
GP: General practitioner
HRQoL: Health Related Quality of Life
hs: High-sensitivity
IOS: Impulse Oscillometry
LoLF: Rate of loss of lung function
MI: Myocardial infarction
ND: Newly diagnosed
NPV: Negative predictive value
OR: Odds ratio
PEF: Peak expiratory flow
PPV: Positive predictive value
ROC: Receiver operator curve
SGRQ: Saint George Respiratory Questionnaire
TIA: Transient ischemic attack
WBC: White blood cell count
